# Impacts of course type and student gender on distance learning performance: A case study in Taiwan

**DOI:** 10.1007/s10639-021-10538-8

**Published:** 2021-04-13

**Authors:** Yu-Chen Hsiao

**Affiliations:** Center for General Education, Chihlee University of Technology, No.313, Sec. 1, Wenhua Rd., Banqiao Dist., New Taipei City, 220305 Taiwan Republic of China

**Keywords:** Distance teaching, Post-COVID19 teaching model, Learning performance, Gender, Course type

## Abstract

The global outbreak of COVID-19 since January 2020 has forced the closure of schools and universities in over 180 countries to control the pandemic, affecting approximately 90% of students worldwide. Distance teaching has been adopted during school closures to suspend classes without suspending learning. Scholars have claimed that distance teaching is more effective than face-to-face teaching and can replace face-to-face courses. However, further investigation is required to confirm whether distance learning is suitable for all types of courses and all students. Thanks to the effective containment of COVID-19 outbreaks in Taiwan, universities in Taiwan face a less problematic situation than do those in other countries; however, plans and preparations remain essential. The present study recruited 18,085 students from a technology university in Taiwan and used the baseline data of the past three academic years before COVID-19 (2016–2018) to explore the influences of course type and gender on distance learning performance. The results revealed that compulsory courses are more suitable for distance learning courses, whereas face-to-face teaching is more suitable for elective and general education courses. The learning performance of males and females is also different: face-to-face courses are more suitable for males, whereas no significant difference between teaching methods was observed in females. This result suggests that not all courses offered by the university are suitable for distance learning courses, and not all students are adept at distance learning. Based on these results, it is recommended that a new teaching model be established for the post-COVID-19 era.

## Introduction

The development of information technology has diversified approaches to teaching and learning. Distance learning enables students to learn at their own pace and establish their own personal course timelines, and this greater flexibility of distance learning may allow greater efficacy (Marki et al., 2000; Shanley et al., 2004; Azeiteiro et al., 2015; Lee et al., 2019) and enable students to undertake full-time employment to pay tuition fees (Nistor & Nyer, 2018a, b). Worldwide, distance learning in universities has recently developed rapidly and has experienced exponential growth (Sutton & Nora, 2008; Allen & Seaman, 2010; Moura et al., 2010; Layne et al., 2013). Furthermore, distance learning has been the focus of numerous studies and innovative thinking (Nistor & Nyer, 2018a, b). An increasing number of studies have reported that distance teaching is more effective than physical classroom teaching, and the performance of distance teaching has been positively recognized by many students (Wen & Chang, 2014). Moreover, it is considered that distance learning could replace traditional face-to-face courses (Azeiteiro et al., 2015; Lee et al., 2019).

However, some researchers hold the opposite view and have suggested that students with online learning performed poorly (Brown & Liedholm, 2002) or equivalently (Bernard, et al., 2004; Wagner et al., 2011). Some studies have revealed that students are satisfied with the teaching effectiveness of distance and face-to-face blended courses (Buzzard et al., 2011; Yu, 2011). It is reported that this blended teaching approach strengthens students’ ability to collaborate (Nistor & Nyer, 2018a, b).

Hsiao and Shiao (2018) proposed gender differences in distance learning performance and found that females perform significantly better than male (Gunn et al., 2003; Price, 2006; Rovai & Baker, 2005). Others found no differences between males and females in terms of their learning outcomes in online courses (Astleitner & Steinberg, 2005; Lu et al., 2003; Sierra & Wang, 2002; Yukselturk & Bulut, 2007). Other studies have indicated that course subject (Jaggars, 2012) and class level (Wladis et al., 2014) also affect the outcome in online learning.

The global outbreak of COVID-19 since January 2020 has forced the closure of schools and universities in over 180 countries to control the pandemic, affecting approximately 90% of students worldwide. During school closures, various countries have adopted distance teaching to maintain students’ rights to learn and achieve the goal of suspending classes without suspending learning (UNESCO, 2020). Because the duration of the COVID-19 pandemic is unknown, more and more universities worldwide have announced the implementation of online teaching for the 2020 fall semester as a measure for preventing the spread of the disease. Therefore, online learning has been an overwhelming response to these closures. In other words, online learning is a way of combating the spread of COVID-19.

Many scholars have questioned whether higher education was prepared for the forthcoming digital era of learning (Houlden & Veletsianos, 2020). The prime minister of Bhutan stated that, because not all teachers are tech-savvy and not all students can afford video-streaming equipment, teachers, parents, and students complain about e-learning—they consider distance teaching a challenge and burden (Rinzin, 2020). Wu (2020) reported that universities around the world rapidly adopted online teaching as a major alternative way to respond the COVID-19 pandemic. Many universities initially focused on transitioning content to an online environment, and not necessarily on online pedagogy.

To sum up, due to the tremendous changes in teaching methods in response to this epidemic, students originally in physical on-site courses cannot but switch to online learning. Certainly, it is expected that online courses should maintain and even enhance the teaching quality to ensure student’s learning performance. A common way to evaluate teaching effectiveness in higher education is student evaluation of teaching (SET) (Centra & Gaubatz, 2000; Steif & Dollár, 2009; Yueh et al., 2012). The measurement items are mainly divided into two parts: the teacher's teaching performance, and the student's self-evaluation regarding learning status. Both should be carried out at the end of each semester, and students should be allowed to evaluate each course they have taken.

This school in this study is a business and technology university with 55 years of history. It is located in the metropolitan area in northern Taiwan. Thanks to the effective containment of COVID-19 outbreaks in Taiwan, the school has been running its full-time schedule, with in-person learning uninterrupted, since February 2020 (the second semester of 2019 in Taiwan). In recent years, the school has been committed to the collection of various learning history data of the students. We have also used those basic data to track and analyze real-time student learning, and we have developed appropriate teaching diagnosis or learning support programs. A comprehensive data-based model for improving student learning effectiveness has been established. As the world is adjusting to a new mode of learning through virtual classrooms, which may well become the new normal in the post-COVID-19 era, the use of baseline data to assess the efficacy of online learning and to consider integrating it into routine education in Taiwan is a crucial issue. Specifically, in this study, we investigate students’ performances by comparing online and in-class learning in different course categories and the gender difference in these learning experiences.

The current study used SET of students’ satisfaction with their teacher’s teaching performance and their self-study evaluation (Yang & Cornelious, 2005; Crawford-Ferre & Wiest, 2012; Yueh & Liang, 2015), as well as the course score (Whipple, 1987; Xu & Jaggars, 2012; Nyer, 2019). These three variables are collectively referred to as learning performance. Comparative analysis of the learning performances of 18,085 students in distance and physical courses in the past three academic years before COVID-19 (2016–2018) was conducted to understand the influence of course type and gender on student learning performance in distance teaching.

### Purposes

On the basis of the provided research background, the objectives of this study were as follows:

1. To explore the effects of course type on distance learning performance,
2. To explore the effects of gender and course type on distance learning performance, and
3. To propose an appropriate teaching model for the post-COVID-19 era.

## Methods

### Participants

The school curriculum is divided into three categories: compulsory, elective and general education courses. A total of 57 distance courses are offered in the academic years 2016–2018, and they account for 1.08% of the total number of courses in the school. Most of these distance courses were directly transcribed into videos from physical courses. The number of students enrolled is 3,617. According to faculty, department, gender, and face-to-face courses of the same type, these 3,617 students were paired in a ratio of 1:4 with 14,468 students who took face-to-face courses. Finally, comparative analysis of the total 18,085 students was performed. The results are presented in Table 1.

**Table 1:** Number of students taking distance learning courses and those taking face-to-face courses

Course Type	Number of Distance Learning Course Students	Number of Face-to-Face Course Students
General Education	1,563	6,252
Elective Courses	1,086	4,344
Compulsory Courses	968	3,872
Total	3,617	14,468

### Dataset

The data of the 18,085 students were collected from the research database of the university’s institutional research office. These data included the students’ demographics such as faculty, department, and gender; the names of the courses they took; teaching satisfaction scores they obtained in those courses; their self-learning satisfaction scores; and their course scores.

### Data analysis

The learning performance scores were expressed using mean and standard deviation (SD). Courses were delivered either online or face-to-face. The Student’s *t* test was adopted to compare the differences in learning performance between the two groups that learned from the different teaching methods. Multiple regression analysis with control variables and the explanatory variable (teaching method) was used to investigate the effects of distance teaching and face-to-face teaching on learning performance.

## Results and discussion

### The effects of course type on distance learning performance

The learning performances of students in the two groups were examined according to course type, and the Student’s *t* test was used to compare the results (Model 1 in Table 2). Regarding students’ learning performance in compulsory courses, scores for the two items—teaching satisfaction and self-learning satisfaction—were not significantly different between the two teaching methods, but significant differences were observed in course scores (P < 0.0001). The results indicated that students’ course scores were significantly higher with distance teaching than with face-to-face teaching. Significant differences were observed between the two teaching methods for all three items of learning performance in elective courses (P = 0.0197, P < 0.0001, P < 0.0001). Our results indicated that face-to-face teaching was more effective than distance teaching. For general education courses, no significant differences in teaching satisfaction or self-learning satisfaction were noted between the two teaching methods. However, students receiving face-to-face teaching had significantly higher course scores than those receiving distance teaching (P < 0.0001).

**Table 2:** Model 1: Comparative analysis of student learning performance in distance learning and face-to-face courses under course type

Learning Performance	Type of Course	Face-to-Face Course		Distance Learning Course		*P *value
		*n*	Mean ± SD	*n*	Mean ± SD	
Teaching Satisfaction	Compulsory	3685	4.18 ± 0.79	812	4.23 ± 0.79	0.1036
	Elective	3908	4.30 ± 0.72	972	4.24 ± 0.70	0.0197
	General Education	5791	4.34 ± 0.67	1449	4.37 ± 0.69	0.1805
Self-learning Satisfaction	Compulsory	3685	3.67 ± 0.71	812	3.63 ± 0.79	0.1299
	Elective	3929	3.77 ± 0.68	972	3.66 ± 0.74	< 0.0001
	General Education	5851	3.69 ± 0.68	1449	3.69 ± 0.79	0.8075
Course score	Compulsory	3872	72.90 ± 20.84	968	77.59 ± 16.78	< 0.0001
	Elective	4344	76.34 ± 19.02	1086	75.31 ± 18.04	< 0.0001
	General Education	6252	80.37 ± 17.26	1563	76.43 ± 22.66	< 0.0001

*SD* Standard deviation

According to these results, compulsory courses appear to be more suitable for distance learning courses, possibly due to students attaching greater importance to compulsory courses, and online teaching materials could be provided to students for review before and after class, which positively influences students’ learning effectiveness. Therefore, online delivery of compulsory courses results in higher course scores. Elective courses and general education courses appear to be more suitable for face-to-face teaching.

Subsequently, multiple regression analysis was performed with teaching method as the main explanatory variable. The correlation between students’ learning performance and teaching method (online or face-to-face) was examined. The results are provided in Model 2 in Table 3.

**Table 3:** Model 2: Factors influencing student’s learning performance in distance learning and face-to-face courses

Learning Performance	Control Variable	Estimate	SD	Standardized Estimate	*t*-value	*P* value
Teaching Satisfaction	Teaching Method					
	Distance vs. Face-to-Face	0.007	0.014	0.004	0.52	0.6031
	Gender					
	Female vs. Male	-0.045	0.012	-0.028	-3.68	0.0002
	Course Type					
	Elective vs. Compulsory	0.106	0.015	0.067	7.10	< 0.0001
	General vs. Compulsory	0.162	0.014	0.112	11.91	< 0.0001
Self-Learning Satisfaction	Teaching Method					
	Distance vs. Face-to-Face	-0.045	0.014	-0.025	-3.26	0.0011
	Gender					
	Female vs. Male	-0.090	0.012	-0.058	-7.44	< 0.0001
	Course Type					
	Elective vs. Compulsory	0.091	0.015	0.058	6.21	< 0.0001
	General vs. Compulsory	0.034	0.013	0.024	2.52	0.0118
Course score	Teaching Method					
	Distance vs.Face-to-Face	-0.755	0.352	-0.016	-2.15	0.0317
	Gender					
	Female vs. Male	5.453	0.307	0.130	17.75	< 0.0001
	Course Type					
	Elective vs. Compulsory	1.825	0.374	0.044	4.87	< 0.0001
	General vs. Compulsory	5.472	0.346	0.141	15.80	< 0.0001

The teaching satisfaction score was non-significantly correlated with teaching method. The scores for self-learning satisfaction and course score for distance learning were significantly lower than those for physical teaching (P = 0.0011, P = 0.0317). That is, after gender and course type were controlled for, teaching method was a crucial factor influencing the self-learning satisfaction and course score items of learning performance. In these two items, students had significantly higher performance in face-to-face courses than in distance learning courses. These results indicated that face-to-face courses are more appropriate for students.

Model 2 also revealed that gender was a factor that influenced learning performance when teaching method and course type were controlled for. Females had significantly lower scores than their counterparts in the teaching satisfaction (P = 0.0002) and self-learning satisfaction (P < 0.0001) dimensions, but they had significantly higher course scores (P < 0.0001) than males. This might be attributed to females having higher expectations of themselves and their teaching satisfaction. In addition, females also had significantly higher course scores than males.

**Fig. 1:**
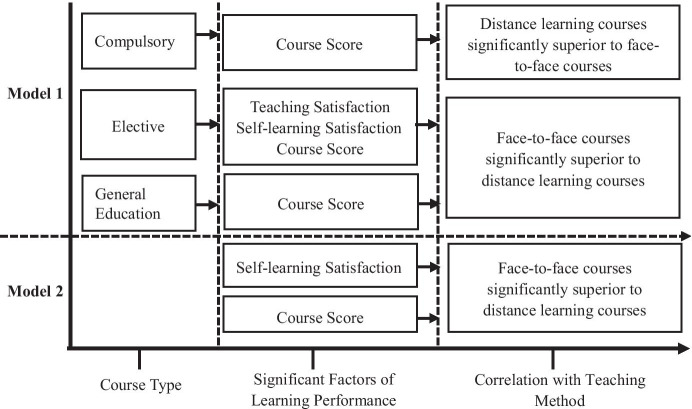
Diagram summarizing the effects of course type on learning performance during distance teaching and face-to-face teaching

A summary of Models 1 and 2 that illustrates the effects of course type on distance learning and face-to-face learning performance is provided in Fig. 1.

### The effects of gender on distance learning performance

Gender is a key factor that affects student’s learning performance (Table 3). To determine the specific gender differences in learning effectiveness, this study performed stratification by gender and compared learning performance according to the two teaching methods. In this section, the performances of 5,450 males and 12,635 females were analyzed (Table 4). The analysis results of Model 3 are provided in Table 5.

**Table 4:** Stratification by gender: Number of students taking face-to-face or distance-learning courses

Gender	Male		Female	
Course Type	Face-to-Face	Distance Learning	Face-to-Face	Distance Learning
Compulsory	1,352	338	2,521	630
Elective	1,140	285	3,205	801
General	1,868	467	4,382	1,096
Total	4,360	1,090	10,108	2,527

**Table 5:** Model 3: Comparative analysis of student learning performance in distance learning and face-to-face courses when stratified by gender and course type

Gender	Learning Performance	Course Type	Face-to-Face Course		Distance Learning Course		
				Mean ± D	*n*	Mean ± SD	*P* value
Male	Teaching Satisfaction	Compulsory	1257	4.19 ± 0.87	237	4.22 ± 0.87	0.5943
		Elective	994	4.34 ± 0.76	241	4.33 ± 0.71	0.8634
		General	1693	4.38 ± 0.73	426	4.40 ± 0.74	0.5610
	Self-Learning Satisfaction	Compulsory	1257	3.71 ± 0.78	237	3.56 ± 0.84	0.0093
		Elective	995	3.85 ± 0.73	241	3.61 ± 0.78	< 0.0001
		General	1704	3.81 ± 0.73	426	3.72 ± 0.87	0.0403
	Course Score	Compulsory	1352	70.08 ± 22.41	338	74.52 ± 17.73	0.0001
		Elective	1140	72.31 ± 21.03	285	69.84 ± 21.00	0.0764
		General	1868	76.24 ± 19.55	467	72.25 ± 23.82	0.0009
Female	Teaching Satisfaction	Compulsory	2428	4.17 ± 0.74	575	4.23 ± 0.76	0.0933
		Elective	2914	4.29 ± 0.70	731	4.21 ± 0.70	0.0088
		General	4098	4.33 ± 0.65	1023	4.36 ± 0.67	0.2197
	Self-Learning Satisfaction	Compulsory	2428	3.65 ± 0.68	575	3.66 ± 0.77	0.9287
		Elective	2934	3.74 ± 0.67	731	3.68 ± 0.73	0.0205
		General	4147	3.65 ± 0.66	1023	3.68 ± 0.75	0.2252
	Course Score	Compulsory	2520	74.41 ± 19.78	630	79.24 ± 16.03	< 0.0001
		Elective	3204	77.77 ± 18.04	801	77.25 ± 16.45	0.4357
		General	4384	82.13 ± 15.87	1096	78.21 ± 21.92	< 0.0001

*SD* Standard deviation

Male’s scores for teaching satisfaction in compulsory, elective, and general education courses did not differ significantly between the distance learning and face-to-face methods; however, the scores for self-learning satisfaction in these three types of courses were significantly higher when the courses were taught by face-to-face than by online delivery (P = 0.0093, P < 0.0001, P = 0.0403). Females’ scores for teaching satisfaction and self-learning satisfaction in elective courses were significantly higher when this type of course was taught face-to-face than when it was delivered online (P = 0.0088, P = 0.0205). No significant difference was observed for the other two types of courses. The course scores of male and female students in compulsory and general education courses differed significantly between the two teaching methods. Their course scores were significantly higher when compulsory courses were taught online than when they were taught face-to-face (P = 0.0001, P < 0.0001). When general education courses were delivered face-to-face, students’ course scores were significantly higher than when these courses were taught remotely (P = 0.0009, P < 0.0001).

This result was similar to the analysis results of Model 1. From the above analytic results, the learning performance of students was better in distance compulsory courses than in physical compulsory courses, while in elective and general courses, learning performance was better in physical courses than in distance courses. These results were in agreement with Coleman and Fararo (1992), in which the rational choice theory was emphasized. Online teaching materials can be provided to students for both pre-class preparation and after-class review, which in turn enhance the effectiveness of students' learning. Therefore, the compulsory courses are taught remotely, and students' course results are better. In addition, Xenos et al. (2002) also found that students may choose the required major for on online courses, and switch elective and general courses to take physical learning. It seems obvious that compulsory courses are more suitable for distance teaching, while elective and general courses are more suitable for face-to-face teaching.

Subsequently, teaching method was set as the main explanatory variable, and course type was controlled for. The correlations between the learning performances of the two genders and teaching method (online or face-to-face) were examined (Model 4 in Table 6).

**Table 6:** Model 4: Factors influencing student’s learning performance stratified by gender

Gender	Learning Performance	Control Variable	Estimate	SD	Standardized Estimate	*t*-value	*P* value
Male	Teaching Satisfaction	Teaching Method					
		Distance vs. Face-to-Face	0.017	0.029	0.009	0.59	0.5523
		Course Type					
		Elective vs. Compulsory	0.144	0.030	0.080	4.80	< 0.0001
		General vs. Compulsory	0.188	0.026	0.119	7.13	< 0.0001
	Self-Learning Satisfaction	Teaching Method					
		Distance vs. Face-to-Face	-0.146	0.028	-0.074	-5.17	< 0.0001
		Course Type					
		Elective vs. Compulsory	0.119	0.029	0.067	4.04	< 0.0001
		General vs. Compulsory	0.114	0.026	0.074	4.44	< 0.0001
	Course Score	Teaching Method					
		Distance vs. Face-to-Face	-0.976	0.711	-0.019	-1.37	0.1701
		Course Type					
		Elective vs. Compulsory	0.848	0.755	0.018	0.26	0.2615
		General vs. Compulsory	4.480	0.671	0.105	6.68	< 0.0001
Female	Teaching Satisfaction	Teaching Method					
		Distance vs. Face-to-Face	0.003	0.016	0.002	0.19	0.8525
		Course Type					
		Elective vs. Compulsory	0.089	0.017	0.059	5.23	< 0.0001
		General vs. Compulsory	0.150	0.016	0.107	9.44	< 0.0001
	Self-Learning Satisfaction	Teaching Method					
		Distance vs. Face-to-Face	-0.007	0.016	-0.004	-0.44	0.6566
		Course Type					
		Elective vs. Compulsory	0.076	0.017	0.052	4.54	< 0.0001
		General vs. Compulsory	-0.001	0.016	-0.001	-0.08	0.9354
	Course Score	Teaching Method					
		Distance vs. Face-to-Face	-0.660	0.399	-0.015	-1.66	0.0979
		Course Type					
		Elective vs. Compulsory	2.295	0.427	0.059	5.37	< 0.0001
		General vs. Compulsory	5.969	0.401	0.163	14.89	< 0.0001

*SD* Standard deviation

From the results of Model 4 in Table 6, it was found that among the three learning performances, only males’ average score for self-learning satisfaction was significantly related to the teaching method, and the average score of distance teaching was significantly lower than that of physical teaching (P < 0.0001). Apparently, when the type of course was controlled for, the teaching method was a crucial factor in the self-learning satisfaction of male students, where physical teaching was significantly better than remote teaching. These findings suggest that male students might be more suitable for physical courses. On the other hand, there was no significant difference in the learning performances of female students under these two teaching methods. This also implied that female students performed better than male students in online learning. These results might be due to females being better at online communication and online course time management than males (McSporran & Young, 2001). This finding was in agreement with a previous study, in which male students found it more difficult to adapt to online learning than females did (Xu & Jaggars, 2014).

This result was similar to the analysis results of Model 2 in several ways. First, the scores for teaching satisfaction had no significant correlation with teaching method, probably due to students’ evaluations of teaching being non-significantly correlated with learning, as observed by Uttl et al. (2017). In addition, students generally complete evaluations in an optimistic and reserved manner because of social expectations or their defense mechanism, causing discrepancy between the study results and reality (Yueh & Liang, 2015). This difference warrants further exploration.

Second, teaching method was the factor influencing self-learning satisfaction, and students had significantly higher performance in face-to-face courses than in distance learning courses. That is, students perceive a more positive learning attitude in face-to-face courses, which implies that face-to-face courses are more suitable for students. Regarding this aspect, several researchers have indicated that the key to online learning is that learners have a higher level of autonomy. However, because of a lack of supervision by teachers, online learners must be able to initiate learning on their own (Bambara et al., 2009; Eisenberg & Dowsett, 1990; Liu et al., 2007; Mupingo et al., 2006). Therefore, if schools intend to implement online learning in the future, they must enhance students’ independent learning capability to promote effective learning. This consideration also suggests that most students prefer face-to-face courses, as evidenced by the significantly higher performance in face-to-face classes than in distance learning courses.

**Fig. 2:**
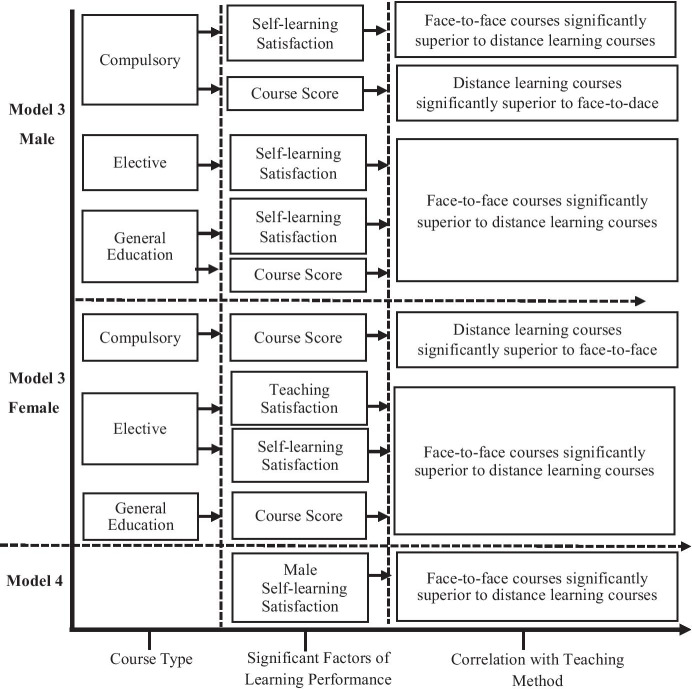
Diagram summarizing the effects of gender on learning performance during distance and face-to-face teaching

A summary of Models 3 and 4 that illustrates the effects of gender on distance and face-to-face learning performance is provided in Fig. 2.

## Conclusion and recommendations

This study explored the effects of course type and gender on learning performance during distance teaching to establish a new teaching model for the post-COVID-19 era. The aforementioned results highlight that, in terms of student learning performance in the three types of courses offered by the university in this study, course type and gender exhibited different influences on learning performance in distance teaching. Compulsory courses are more suitable for distance learning courses, whereas face-to-face teaching is more suitable for elective and general education courses. In addition, Face-to-face courses are more suitable for males, whereas no significant difference between teaching methods was observed in females. Students (particularly male students) tended to be passive in learning, and their independent learning ability may require further improvement. This result suggests that not all courses offered by the university are suitable for distance learning courses, and not all students are adept at distance learning. Therefore, schools are unlikely to replace face-to-face courses with distance learning courses, and online delivery of courses does not meet the needs of students.

This study had several limitations. Among the three factors in learning performance, the course scores were not uniformly standardized. In future analyses, a more suitable standardized score should be employed for investigation. In addition, students’ assessment of teaching effectiveness is an integral part of SET. In this study, except for elective courses, which were significantly correlated with teaching method in the students’ evaluation of teaching satisfaction, and for which face-to-face courses were superior to distance learning courses, the other types of courses had no significant correlation with teaching method. This finding is not consistent with experiences or the results of numerous other studies and, therefore, merits further exploration to achieve the goal of using SET to improve teaching quality. Furthermore, this research was based on the analysis of baseline data collected before the COVID-19 pandemic. If influenced by the pandemic, students’ preferences for courses may have changed. In view of the possible differences in the academic performance and course satisfaction of students taking distance and physical courses before and after the COVID-19 pandemic, further comparative analysis may be worthwhile. For reproducibility, the survey data is available upon request.

However, as the world is adjusting to a new mode of learning through virtual classrooms, integrating the efficacy of online learning into routine education in schools is important. We propose five recommendations on the basis of the current results. These recommendations could help schools devise a new teaching model for the post-COVID-19 era and establish smart campuses where digital learning is the norm.

1. More online learning courses can be introduced. A total of 57 distance courses are offered in the academic years 2016–2018, and they account for 1.08% of the total number of courses in the school. The number of online learning courses is insufficient. The forms of digital courses are very diverse, including distance courses, hybrid courses, flip courses, and MOOCs courses. It is recommended that school opens various types of courses to provide students with more opportunities for online learning.
2. External Massive Open Online Courses (MOOCs) should be introduced. Students can be encouraged to take MOOCs offered in Taiwan or abroad. Engage with other teachers and students in higher education worldwide, and bolster their competitiveness.
3. The quality of online teaching materials can be improved. Online teaching materials are a critical factor in ensuring the effectiveness of any form of online teaching. Most of these distance courses opened in the school were directly transcribed into videos from physical courses. It is not necessarily a curriculum designed according to digital learning methods, which may affect students' interest and effectiveness in learning. Schools could consider employing digital learning professionals who can help teachers to design online courses and materials. In addition, schools should make use of learning systems and provide students with real-time learning assessments to increase students’ learning motivation and foster students’ independent learning capability.
4. Schools should have all necessary online learning equipment. It is suggested that schools install a variety of software and hardware equipment and actively assist students and teachers in familiarizing themselves with the operation and application of various technologies, thereby improving the effectiveness of online teaching and learning.
5. Online learning styles should be investigated. The learning process is highly individualized. The results show that not all students are adept at distance learning. School should investigate online learning styles and determine learners’ individual learning styles and preferences to provide suitable instruction. Then, gradually guides all students to have experiences in taking online learning courses, as to establish a digitized personal adaptive learning mechanism.

## Data Availability

Data is available upon request from the corresponding author.
